# Dynamic interplay between a TonB-dependent heme transporter and a TonB protein in a membrane environment

**DOI:** 10.1128/mbio.01781-24

**Published:** 2024-10-30

**Authors:** Kamolrat Somboon, Oliver Melling, Maylis Lejeune, Glaucia M. S. Pinheiro, Annick Paquelin, Benjamin Bardiaux, Michael Nilges, Phillippe Delepelaire, Syma Khalid, Nadia Izadi-Pruneyre

**Affiliations:** 1 School of Chemistry, University of Southampton, Southampton, United Kingdom; 2 Institut Pasteur, Université Paris Cité, CNRS UMR3528, Structural Bioinformatics Unit, Paris, France; 3 Institut Pasteur, Université Paris Cité, CNRS UMR3528, Bacterial Transmembrane Systems Unit, Paris, France; 4 Institut de Biologie Physico-Chimique, UMR 7099, CNRS Université de Paris, Paris, France; 5 Department of Biochemistry, University of Oxford, Oxford, United Kingdom; Fred Hutchinson Cancer Center, Seattle, Washington, USA; University of Maryland Baltimore, Baltimore, Maryland, USA

**Keywords:** bacterial membrane, energy transduction, ton system, heme transport

## Abstract

The envelope of Gram-negative bacteria is composed of two membranes separated by the periplasmic space. This organization imposes geometrical and distance constraints that are key for the mechanism of action of multicomponent systems spanning the envelope. However, consideration of all three compartments by experimental approaches is still elusive. Here, we have used the state-of-the-art molecular dynamics simulation in an *Escherichia coli* envelope model to obtain a dynamic view of molecular interactions between the outer membrane heme transporter HasR and the inner membrane TonB-like protein HasB. Their interaction allows the transfer of the inner membrane proton-motive force derived energy to the transporter for heme internalization. The simulations that incorporate both membranes show the key role of periplasmic domains of both proteins and their dynamics in complex formation and stability. They revealed a previously unidentified network of HasR-HasB protein-protein interactions in the periplasm. Experimental validation (mutations, *in vivo* phenotypic and biophysical assays) provides support for the simulation-predicted interactions. Based on structural and sequence conservation, the network of interaction revealed in this study is expected to occur in other nutrient import systems.

**IMPORTANCE:**

Gram-negative bacteria import scarce nutrients such as metals and vitamins by an energized mechanism involving a multicomponent protein system that spans the cell envelope. It consists of an outer membrane TonB-dependent transporter (TBDT) and a TonB complex in the inner membrane that provides the proton motive force energy for the nutrient entry. Despite the intense research efforts focused on this system (a) from structural and fundamental microbiology perspectives and (b) for the interest in the development of new antibacterial strategies, the molecular mechanism of the system is not at all well understood. The lack of understanding comes from incomplete structural data and the experimental difficulties of studying an inherently flexible multicomponent complex that resides within the heterogeneous environment of the double membrane bacterial cell envelope. To address these challenges and obtain a comprehensive view of the molecular interactions at atomic level, here, we have used the combined power of advanced molecular simulations and complementary microbiology and biochemical experiments. Our results represent a significant step forward in understanding the structural and molecular bases of this vital mechanism.

## INTRODUCTION

Gram-negative bacteria are delimited by an envelope composed of two membranes [an outer membrane (OM) and an inner membrane (IM)] separated by the periplasmic space. The IM TonB complex and the specific OM TonB-dependent transporters (TBDTs) are used to import scarce and essential nutrients, such as metal sources, vitamin B_12_, specific carbohydrates, etc (1, 2). As both the OM and the periplasm lack an energy source, the TonB complex is used to couple substrate entry to the proton- motive force provided by the proton gradient across the IM. Due to this organization, this system is an ideal model for understanding the mechanism of signal and energy transfer through the bacterial cell envelope.

The TonB complex comprises three IM proteins, TonB, ExbB, and ExbD. TonB is the only protein of the complex that directly interacts with the TBDT and thus must be responsible for energy transfer, whereas ExbB and ExbD form a proton channel (3). The structures of the complete TonB complex and the full-length TonB protein are still unknown (4). TonB is composed of an N-terminal transmembrane helix inserted into the IM, an unstructured proline-rich domain in the periplasm followed by a C-terminal and globular domain that is the only identified domain of interaction with TBDT. Only the structure of this globular periplasmic domain of TonB, either alone or in complex with TBDT, has been reported (5–9). TonB interacts with the TonB-box, a stretch of 7–10 conserved residues that is exposed to the periplasm and located at the N-terminus of TBDT (7, 8). It is proposed that, upon this interaction and in the presence of energy, rotation of the proton channel (comparable to the Mot flagellar system) (10) and/or a pulling movement of TonB induce conformational changes in the TBDT, eventually opening a channel for nutrient entry. This energizing mechanism is not well understood despite being the focus of numerous structural and functional studies. There are several challenges for the study of these systems, including lack of structural data for the complete system, the inherent flexibility of such a large system, its energized nature, and its organization through the three compartments of the cell envelope. Consequently, experimentally derived mechanistic details of the whole envelope-spanning system are still elusive.

To overcome the experimental challenges in studying such trans-envelope systems, here, we used a combination of *in silico*, *in vivo*, and *in vitro* approaches. We employed molecular modeling and molecular dynamics simulations in an *Escherichia coli* cell envelope model to decipher the network of interactions among a TBDT, the OM heme transporter (HasR), and an IM TonB-like protein (HasB). These two proteins are part of the Has system (heme acquisition system) developed by commensal and pathogenic bacteria to acquire heme, the major iron source in mammalian hosts (11). The system studied here is from *Serratia marcescens* and functionally reconstituted in *Escherichia coli* (4, 12). HasR functions in synergy with a hemophore (HasA) that harvests extracellular heme, either free or bound to host hemoproteins (*e.g.,* hemoglobin). Through protein-protein interaction, the hemophore transfers its heme to HasR for internalization. The HasA-HasR complex then dissociates, and HasA is recycled into the external medium (13).

HasR displays a similar barrel and plug structural organization to other TBDTs whose structures are known—it has a 22-stranded β-barrel forming a transmembrane domain anchored in the OM, with 11 long extracellular loops and 11 shorter periplasmic loops (14). HasR belongs to a class of TBDTs coupled to a signaling activity (15). In these TBDTs, the TonB-box is not at the extreme N-terminus but is connected to a periplasmic globular domain called the signaling domain (here abbreviated as HasR_SD_) (16). HasR_SD_ signals the presence of extracellular sources of heme (heme + HasA) to an anti-σ/ECFσ factor pair that activates the expression of the *has* operon (17). The energy provided by the TonB complex is required for both signaling and transport activities of HasR (15, 17, 18).

Previously, we combined the X-ray structure of HasR (14) and the nuclear magnetic resonance (NMR) structure of HasR_SD_ with electron microscopy (EM) and SAXS data to obtain a structural model of the full-length HasR in its free form (apoHasR) and in complex with HasA and heme (holoHasR). This model showed for the first time that the signaling domain of a TBDT is projected far from the β-barrel (at 8 and 7 nm in the apo- and holo-forms, respectively) *via* a flexible linker (10). A similar positioning of the signaling domain with respect to the β-barrel was further shown in the X-ray structure of FoxA from *Pseudomonas aeruginosa* (9).

In a previous study, we solved the structure of the periplasmic C-terminal domain of HasB (HasB_CTD_) (19) and studied its interaction with a peptide containing the TonB-box of HasR (G_95_ALALDSL_102_). We showed that a salt bridge formed between HasR (D_100_) and HasB (R_169_) is crucial for both maintaining the interaction between the proteins and for HasR function. Furthermore, by using isothermal titration microcalorimetry (ITC), we showed that the presence of heme and HasA modulates the network of interactions between HasR and HasB (20). However, the precise nature of the inter-protein interactions, their variations, and their impact on the conformational dynamics of the proteins are yet to be completely characterized.

In this work, molecular dynamics (MD) simulations of the HasR-HasB complex in apo- and holo-forms, *i.e.*, in the presence of heme and HasA (holoHasR-HasB), revealed previously unidentified networks of interactions and the key role of specific HasR periplasmic loops in complex formation and stability. Simulations of the complex in two periplasm models of differing widths confirmed the stability of these interactions and showed the conformational flexibility of the proline-rich domain of HasB. We validated the predicted network of interactions by mutations and *in vivo* phenotypic and *in vitro* biophysical assays. Interestingly, we showed that the HasR-HasB interactions are modulated by the external signal, i.e., the presence of heme and HasA bound to HasR. This work represents the first example in which atomistic level details are uncovered of the dynamic interplay between a TBDT and a TonB-like protein with inclusion of both membranes of the cell envelope.

## RESULTS

### Molecular dynamics of the HasR-HasB complex

**Fig 1:**
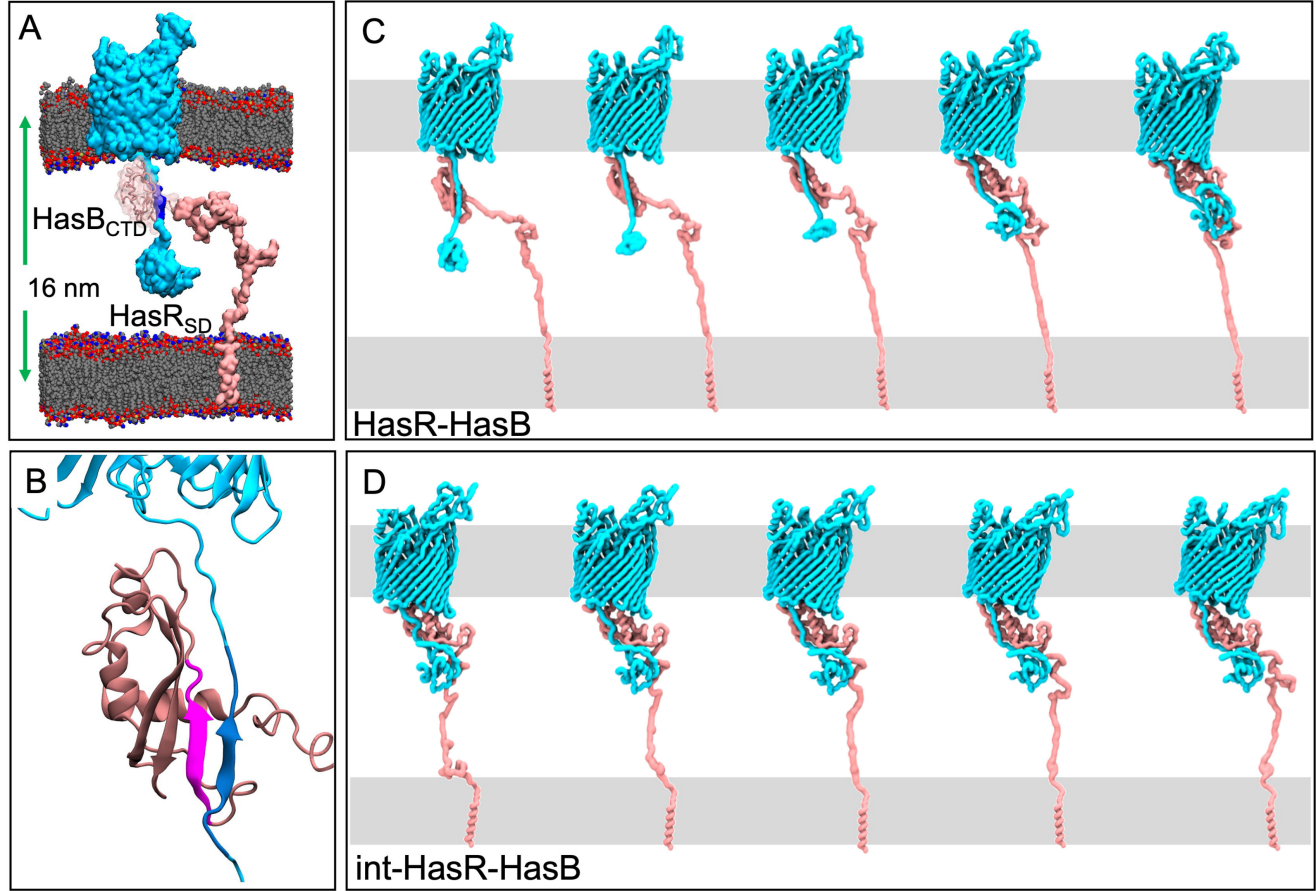
Motion of the HasR-HasB complex. (A) Initial model of the HasR-HasB complex at time = 0. Each protein is embedded in a model membrane. The membrane lipids are gray, red, and blue; HasR is cyan, its TonB-box is blue, HasB is pink, and water and ions are omitted for clarity. Proteins are shown in surface representation, while the membranes are shown as spheres. (B) A close-up view of the inter-protein β-sheet in the original model. The strand from HasR is blue, and the strand from HasB is magenta. (C) Projection along the first principal component from the initial HasR-HasB simulation from which we extracted the int-HasR-HasB configuration. (D) Projection along the first principal component from an int-HasR-HasB simulation

We started with the compositionally simplest system (HasR-HasB) consisting of HasR in its apo-form (without its extracellular substrates) and HasB (Fig. 1). We constructed an initial model of these two proteins each in a model membrane based on their structural organization and guided by our previous experimental findings, as described in the Supplementary Information (Fig. 1A and B). The IM is modeled as a 1-palmitoyl-2-oleoyl-sn-glycero-3-phosphoethanolamine (POPE) bilayer. The OM has a lower leaflet of 100% POPE but an upper leaflet of 1,2-dilauroyl-sn-glycero-3-phosphocholine (DLPC).

In the HasR model, the periplasmic linker was almost fully extended at a distance of 11.5 nm from the barrel. The HasB_CTD_ is located at ~6.8 nm from the HasR barrel (center of mass measurements). The HasB transmembrane domain is a single helix located in the model IM. The two membranes are separated by ~12 nm, and this is the shortest distance (the distance from the center of the OM to the center of the IM is ~16 nm). This was deemed to be a good compromise between the average of the periplasmic thicknesses reported in the literature and keeping the simulation system size practical (21) (Fig. 1A).

### Visual analyses and subsequent system setup

In one of the two simulations of the initial HasR-HasB model as described above, we observed interaction of two periplasmic loops of HasR with HasB_CTD_ (discussed in detail later). Therefore, we initiated two additional simulations of the HasR-HasB complex from a point at which the contact between the loops of HasR and HasB has already been established (int-HasR-HasB). It is useful here to list all of the simulations studied in this work given that a number of different systems were simulated, and a summary is also provided in Table 1. Overall, we simulated the HasR-HasB complex as (1) HasR-HasB in which wild types of both proteins are considered in the original model, with HasR in the apo-form (2 × 300 ns), (2) int-HasR-HasB in which the simulations are initiated after the contact between HasB and the periplasmic loops of HasR has been made (4 × 300 ns) in the original HasR-HasB simulations, (3) HasR_L4_-HasB in which the L4 periplasmic loop of HasR is mutated as described below (2 × 200 ns), (4) holoHasR-HasB in which heme and HasA are included in the complex (2 × 300 ns), (5) HasR-HasB_wide_ or the HasR-HasB system in which the periplasmic space is extended to a width of 23 nm (4 × 50 ns), and (6) the _LPS_HasR in which only HasR in a membrane composed of 100% LPS in the outer leaflet and a mixture of phospholipids (see Table 1 for details) in the inner leaflet of the lipid bilayer (2 × 500 ns) is contained.

**TABLE 1:** Summary of the equilibrium MD simulation systems

System	Temperature (K)	Simulation length (ns)
HasR-HasB	303 K	300 (x2)
int-HasR-HasB^*a*^	303 K	300 (x4)
holoHasR-HasB	303 K	300 (x2)
HasR_L4_-HasB	303 K	200 (x2)
HasR-HasB_wide_^*b*^	303 K	50 (x4)
_LPS_HasR^*c*^	303 K	500 (x2)

^*a*^ The int-HasR-HasB simulations were initiated from a structure extracted after 100 ns from one of the HasR-HasB simulations after the contact with L1 and L4 had already been established.

^*b*^ The int-HasR-HasB in the wide periplasm simulations were initiated from the same structure as the int-HasR-HasB simulations, but the inner membrane with HasB helix was translated to be lower by ~8 nm. The proline-rich domain from residues 37 to 140 was remodeled by using Modeller software (two models were generated). To allow the linker regions to relax its structure, we subjected the remainder of protein structure, except the modeled linker region, to position restraints of 1,000 kJ mol^−1^ nm^−2^ for 50 ns using gromos96 54a7 force field (22). The equilibrated protein model was then placed in the double membrane model, followed by a short equilibration with a position restraint of 50 kJ mol^−1^ nm^−2^ on the backbone for 10 ns by using CHARMM36 forcefield.

^*c*^ The _LPS_HasR simulations were built by embedding the HasR barrel and plug domain (Protein Data Bank entry 3CSL) in the outer membrane with the predefined LPS model of *E. coli* in CHARMM-GUI. The lower leaflet of the outer membrane contains POPE, 1-palmitoyl 2-cis-vaccenic phosphatidylglycerol (POPG), and tetraoleoyl cardiolipin (TOCL2) in the ratio 18:1:1 (23, 24), while the upper leaflet of the outer membrane contains a predefined Ra-LPS model of *E. coli* available in CHARMM-GUI (25, 26). The HasR with the Ra-LPS model was then subjected to the equilibration steps shown in the Table S1.

### Secondary structure and structural integrity

Given that the starting conformations of the complexes are models, albeit based on structural data and guided by experimental information, we first evaluated the structural integrity of the folded domains. The root mean square deviation (RMSD) from the initial model and the secondary structures were evaluated. In all cases, the overall fold of the proteins was conserved. The RMSD; root mean square fluctuation (RMSF) of one simulation of each of HasR-HasB, HasR_L4_-HasB, holoHasR-HasB, and HasR-HasB_wide_; and secondary structure analysis of one simulation of HasR-HasB and HasR-HasB_wide_ are provided in the Supplementary Information (Fig. S1).

### Inter-protein motion

The long disordered periplasmic regions of HasR and HasB impart considerable flexibility to the complex in the periplasm. Therefore, we next explored the inter-domain and inter-protein movements. A useful way of filtering the large-scale motions is principal components analysis (PCA). We employed this method to characterize the motion along the first eigenvector for the HasR-HasB complexes in the HasR-HasB and int-HasR-HasB systems (Fig. 1C and D). Interpolation between the extreme conformations sampled in each case shows a distinct pattern in the inter-protein motions. The principal motion in the initial HasR-HasB simulations is a reduction in the distance between the HasR_SD_ and the HasR barrel, as well as a concomitant reduction in the distance between HasB_CTD_ and HasR_SD_ as shown in Fig. 1C. In simulations of the int-HasR-HasB system (Fig. 1D), the major motion is a rearrangement of HasB_CTD_ with respect to the HasR linker and HasR_SD_ (since the large motion of HasR_SD_ and HasB_CTD_ had already occurred prior to the start of this simulation). These data strongly suggest that the HasR periplasmic region (the linker including the TonB-box G_95_ALALDSL_102_) is considerably flexible. A more quantitative analysis of the reduction in the distances is provided in the Supplementary Information (Fig. S2). A striking feature of the measurements is the consistency in the simulations; measurements in all independent simulations stabilize to very similar values for the HasR barrel-HasR_SD_ and HasR barrel-HasB_CTD_ distances (Fig. S2). In particular, for the int-HasR-HasB simulations, the values across the four independent simulations are essentially the same. This indicates that, in terms of large-scale movement, the protein domains have reached a metastable state for the HasR-HasB protein complexes.

### HasB-HasR TonB-box salt bridge

**Fig 2:**
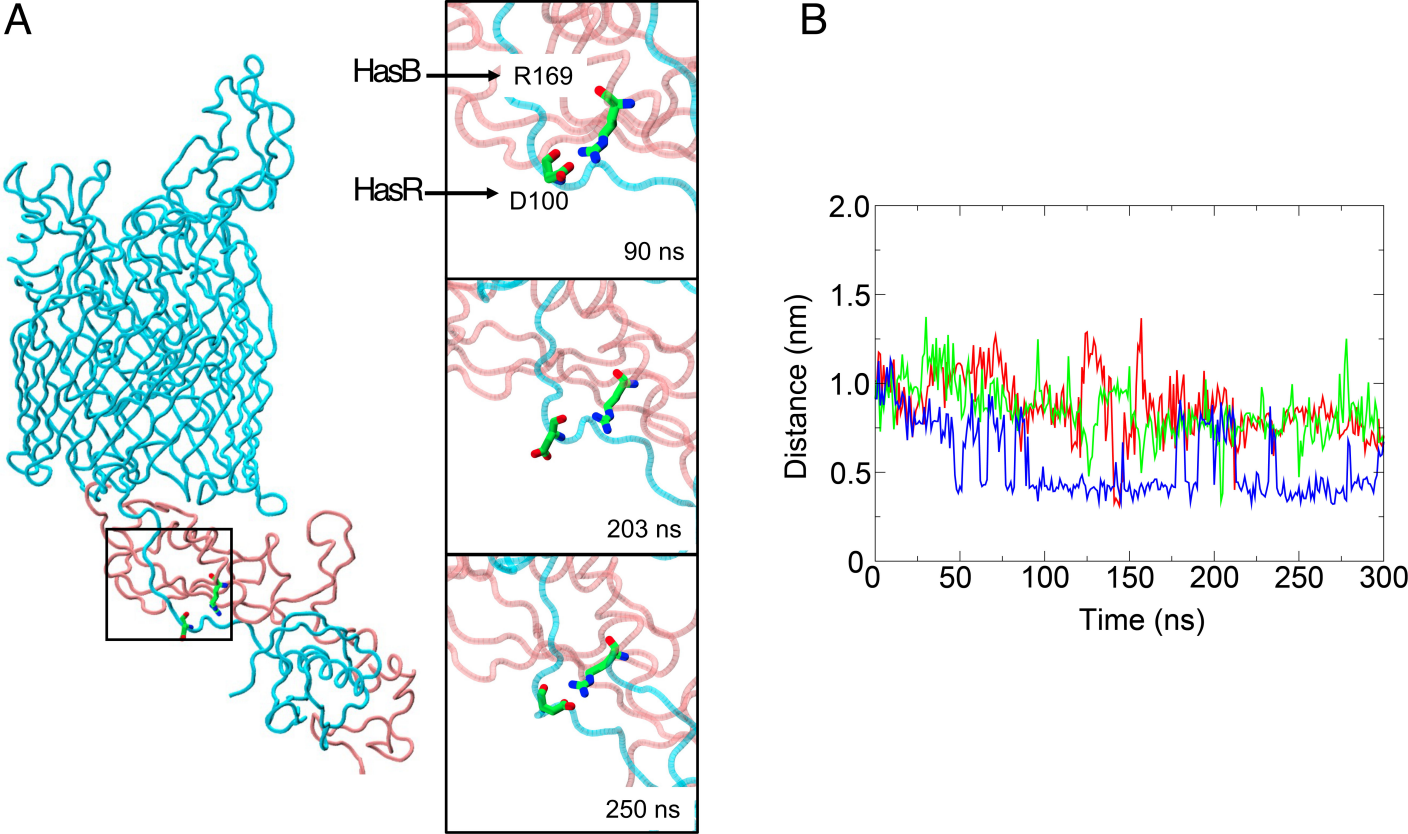
A key salt bridge. (A, left) Snapshot of HasR and HasB from an int-HasR-HasB simulation. The D_100_-R_169_ salt bridge is shown in stick format (HasR and HasB colors as in Fig. 1). The panels on the right show the time course of salt bridge intact (top), disrupted (middle), and reformed (bottom). (B) Minimum distance between D_100_ and R_169_ acidic and basic moieties, respectively, as a function of time from three of the int-HasR-HasB simulations (the salt bridge did not form in the fourth simulation).

In three of the four int-HasR-HasB simulations, we observed spontaneous formation of the salt bridge between D_100_ of the HasR TonB-box and R_169_ of HasB, which was previously identified by our experimental work (19). Interestingly, in some simulations, the interaction is disrupted (defined as the minimum distance between the residues > 0.35 nm) but then reforms either for an extended period or for transitory periods (Fig. 2). The observation that the interaction reforms multiple times over the relatively short timescales accessible to MD simulations demonstrates the persistent nature of the salt bridge.

### HasR-HasB inter-protein β-sheet

**Fig 3:**
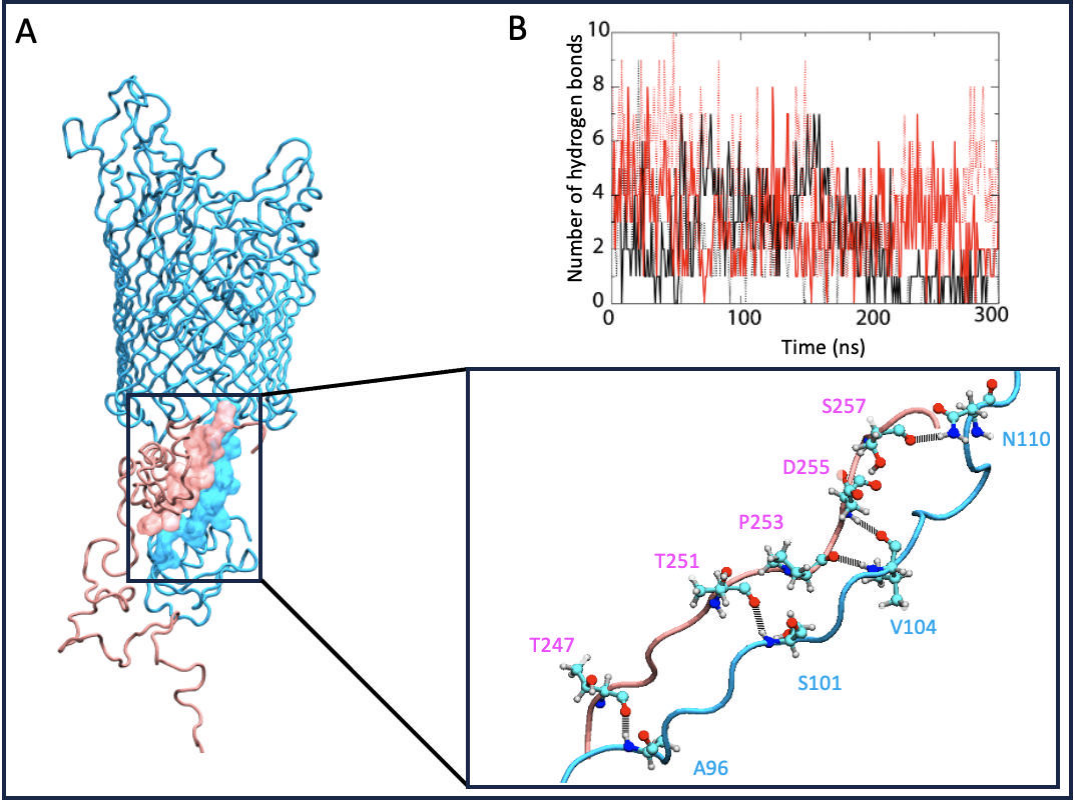
HasR-HasB inter-protein β-sheet. (A) The location of the inter-protein β- sheet is shown in the panel on the left, and the details of hydrogen bonding residues are shown in the panel on the bottom, right. For clarity, only those residues participating in hydrogen bonding in this frame of the simulation are shown explicitly. HasR is cyan, HasB is pink. Atoms are colored as follows: oxygen, red; nitrogen, blue; carbon, cyan; and hydrogen, gray. (B) Number of hydrogen bonds in the inter-protein β-sheet as a function of time from four int-HasR-HasB simulations.

In the HasR-HasB simulations, a network of hydrogen bonds forms between the third β-strand of HasB and a stretch of HasR (residues 93–111) that contains the TonB-box (Fig. 3). The residues of both proteins in these regions can form hydrogen bonds with each other through sidechain and backbone interactions (Fig. 3A). Their orientation and proximity enable the proteins to form an inter protein β-sheet. As some of the simulations proceed, the number of hydrogen bonds in this region decreases (Fig. 3B) and hydrogen bonding between backbone atoms is in some cases replaced by hydrogen bonds between side chains. Thus, this region of interaction is labile and while the number of hydrogen bonds fluctuates, the β-strands remain in close proximity to each other. We note here that an equivalent inter-protein β-sheet was reported in the X-ray structures of the complexes between TonB_CTD_ and the TonB-box of the TBDTs FhuA and BtuB from *E. coli*, as well as FoxA from *P. aeruginosa* (7–9).

### Interaction of HasR periplasmic loops with HasB

**Fig 4:**
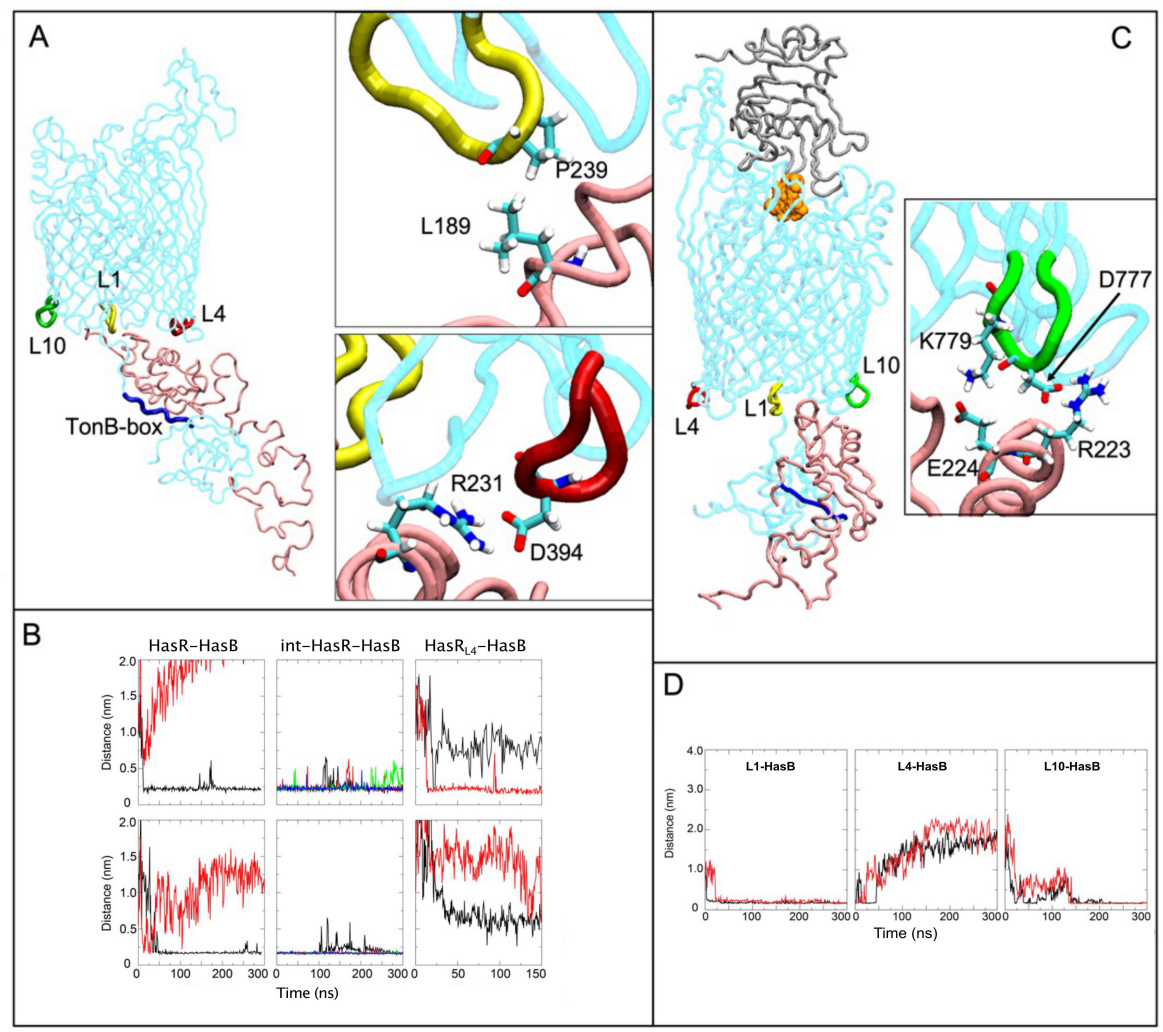
Interaction of HasR periplasmic loops with HasB. (A) HasR loops L1, L4, and L10 (yellow, red, and green, respectively) and the TonB-box (blue) are highlighted. The insets show two key stabilizing interactions between HasR L1 and HasB (top) and HasR L4 and HasB (bottom). (B) The minimum distance between HasR (L1, top row; L4, bottom row) and HasB as a function of time in the two original HasR-HasB simulations (left), the four int-HasR-HasB simulations (middle), and the two HasR_L4_-HasB simulations (right). Each simulation is represented in a different color. (C) HasR L10 and HasB interactions when heme (orange) and HasA (gray) are present. The inset shows two key stabilizing interactions between HasR L10 and HasB. Color code is as in (A). (D) Distances among L1 (left), L4 (middle), and L10 (right) and HasB as a function of time in the holoHasR-HasB simulations.

As already discussed, at the start of the HasR-HasB simulations, there is a large inter-protein motion in which the distance between the HasR_SD_ and the HasB_CTD_ is reduced (Fig. 1C). In one of the simulations, this movement results in interactions of HasB with HasR periplasmic loops L1 and L4, the loop numbering being from the N- to the C-terminus (Fig. 4A). The interactions formed spontaneously within about 20 ns for L1 and 50 ns for L4 and then remained stable for the duration of the simulation (Fig. 4B). The int-HasR-HasB (4 × 300 ns) simulations were initiated from a frame in this trajectory after these loops had already made contact with HasB_CTD_. The L1-HasB and L4-HasB interactions were stable for the duration of all four of these simulations (Fig. 4B).

The HasR L1-HasB interactions are largely mediated by hydrophobic contacts between A_238_ and P_239_ of L1 with L_189_ of HasB_CTD_ (Fig. 4A). Once the interactions with L1 are formed, the HasR linker and HasB_CTD_ move with respect to each other and then HasB_CTD_ contacts L4 *via* several salt bridges. The most prevalent are between HasR L4 residues (E_393_ or D_394_) with either K_230_ or R_231_ of HasB (Fig. 4A) and between E_393_ of HasR and R_223_ of HasB. While the L4-HasB_CTD_ interaction exists for the duration of the int-HasR-HasB simulations, no single salt bridge is observed intact for the full 300 ns duration of any of these simulations. Instead, the salt bridges are observed to form, break, and often reform throughout the simulations, suggesting either a protein-protein interface stabilized by non-specific interactions or that the simulations are not long enough to achieve a stabilized interface between the two proteins. Yet, the persistence in the number of contacts between these regions of the proteins across all simulations suggests that L4 plays a key role in the interaction between HasR and HasB_CTD._ Two additional simulations were performed with a variant of HasR in which L4 (P_392_EDVDWLD) is replaced by the sequence ANATSANA; we later refer to this mutant system as HasR_L4_-HasB. In one simulation, there is no contact between L4 of HasR and HasB and, in the other, L4 contacts HasB only *via* intermittent hydrogen-bonding interaction between N_393_ of L4 and K_184_ or D_185_ of HasB (Fig. S3). Interestingly in neither simulation of the L4 mutant is the salt bridge between D_100_ of HasR TonB-box and R_169_ of HasB maintained. Interaction with L1 is maintained although the binding mode to HasB differs in both simulations (Fig. S3). In summary, mutation of loop L4 of HasR alters the mode of interaction between HasR and HasB.

As the movements of the two proteins occur in the periplasm, which has been reported to range in width from 10 to 50 nm (21, 27), we performed additional simulations using a model cell envelope with a wider periplasm (~23 nm) to assess the robustness of the interactions observed in our simulations. These simulations are explained in full detail in the Supplementary Information (Fig. S4). In summary, these simulations indicate that the HasR-HasB interactions are stable within a wider periplasm and that the HasB proline-rich domain has considerable conformational flexibility and can likely adopt a range of conformations at different stages of its functional mechanism.

Furthermore, we note that the impact of lipopolysaccharide (LPS) on the protein conformational dynamics is neglected here given that we model the outer leaflet of the OM as DLPC phospholipids. The reason for this is due to the slow-moving nature of LPS; simulations of HasR in an OM model containing LPS (without O-antigen) showed very little motion of the protein in 500 ns (Fig. S5).

### Effect of the presence of heme and HasA on the HasR-HasB interaction

The heme internalization through HasR and the ejection of HasA require energy that is provided by TonB/HasB (13). The transduction of the energy likely originates *via* the interaction of TonB/HasB with the transporter HasR, as has been shown for other TBDTs (1, 28, 29). In a previous study, we showed that the presence of heme and HasA on the extracellular side of HasR modified its interaction with HasB (20). Here, to gain insights into this signaling process and to identify the interactions that are modified, we simulated (2 × 300 ns) the HasR-HasB complex in the presence of HasA and heme on the extracellular side of HasR (holoHasR-HasB). In one of these simulations the D_100_-R_169_ salt bridge was remarkably stable. Given the importance of this salt bridge in the heme internalization *via* HasR (19, 20), we analyzed this simulation in further detail.

As in the HasR-HasB simulations, at the early stage, L4 and L1 of HasR (now loaded with HasA and heme) interacted with HasB. The interactions were not present at the start of the simulation but formed spontaneously as the simulation proceeded. Whereas the L1-HasB interaction remained stable during the simulation, the interaction with L4 was eventually lost (Fig. 4D). Instead, loop 10 of HasR (L10) interacted with HasB. This interaction involves D_777_R_778_K_779_ of HasR L10 and R_223_E_224_ of HasB and remained intact during the simulation (Fig. 4C and D). Thus, the MD simulation suggests that the HasR-HasB interaction mode is altered when heme and HasA are present on the extracellular face of HasR. We further explored this hypothesis below with experimental work.

### *In vivo* phenotypic tests to support impacts of HasR loops in the HasR-HasB interaction

To assess the functional importance of HasR L4 and L10, we generated different mutants of these two loops: (i) HasR_L4_ that was tested *in silico* above; (ii) its equivalent mutant of loop 10 called HasR_L10_ in which the residues of loop 10 (R_774_AFDRKLD) were replaced by eight neutral or polar residues (ANATSANA); and (iii) two additional mutants in which the residues of the loop 4 and 10 were replaced by only four neutral and polar residues (ATSA) called HasR_L4short_ or HasR_L10short,_ respectively. Four residues are the minimum required to form a periplasmic loop linking two β-strands and would ensure the integrity of the β-barrel.

First, we evaluated the expression levels of each mutant along with their membrane insertion and surface accessibility. All HasR mutants were expressed at the same level as the wild type (Fig. S6A). Their membrane insertion and accessibility at the surface of whole cells were tested on dot-blot with anti-HasR antibody (Fig. S6B, left panel) and with anti-HasA antibody after incubation with HasA (Fig. S6B, right panel). The dot-blot with anti-HasA antibody was used to assess the HasA binding activity of HasR variants at the cell surface. All the cells that expressed HasR mutants reacted with anti-HasR antibody, showing that all the mutants are well inserted into the membrane and also exposed at the cell surface (Fig. S6B, left panel). With the exception of HasR_L10_ that was highly affected in its capacity to bind HasA, all the other HasR variants retained their capacity to bind HasA at their surface even at the lowest concentration of 1 nM (Fig. S6B, right panel).

**Fig 5:**
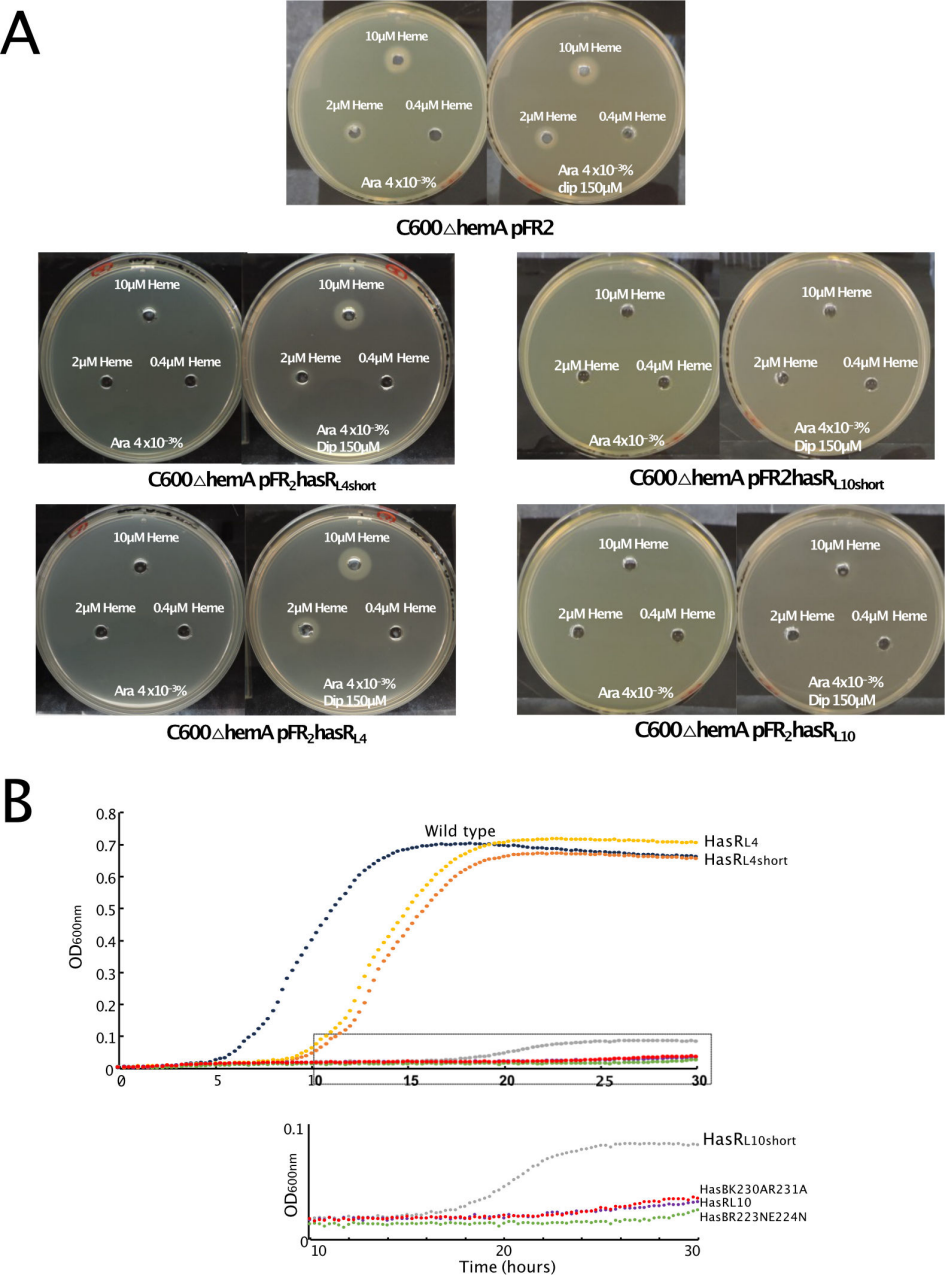
Bacterial phenotypic assays in the presence of HasR and HasB variants. (A) Heme acquisition of HasR variants on solid plate were analyzed with *E. coli* C600*∆hemA* strain harboring pFR2 derivative plasmids for the expression of HasR variants. Bacteria were grown in LB at 37°C to an OD_600nm_ of 1. An aliquot of 100 µL of this culture was mixed with 3.5 mL of soft agar and spread on the top of a Petri dish. Holes were punched in both layers and filled with heme-BSA at 0.4, 2, or 10 µM. The plates were incubated overnight at 37°C and contained 40 µg/mL arabinose, plus or minus 150 µM dipyridyl to induce TonB complex expression. The rectangle in the lower part of the curve is zoomed at the bottom. (B) Growth curves of *E. coli* C600*∆hemA∆exbBD* harboring pBAD24ExbBDsm encoding ExbB and ExbD from *S. marcescens* and pAMHasISRADEB encoding the whole *has* locus with HasR or HasB variants, at 37°C in LB medium supplemented with 100 µM dipyridyl, 4 µg/mL arabinose, and 1 µM heme-BSA, as a heme source. The OD_600nm_ was recorded every 15 minutes for 30 hours.

Next, we performed bacterial growth tests both in liquid and solid media and in different contexts. The heme acquisition of HasR variants was tested on solid medium with *E. coli* C600*∆hemA* strain expressing either HasR wild type or mutants. This *E. coli* strain requires an external source of heme to grow. As the heme source, we added heme-BSA at different concentrations from 0.4 to 10 µM (Fig. 5A). In liquid medium, we monitored the bacterial growth in the context of the whole *has* operon. The presence of the *has* operon enabled us to test the activity of HasR from transcription activation to the HasA-mediated heme entry. For this test, we used *E. coli* C600*∆hemA∆exbBD* strain transformed with two plasmids: one for the expression of the whole Has locus (HasISRADEB) and under the control of its own regulatory elements (15) and the other one allowing the expression of ExbB and ExbD from *S. marcescens* (4).

The bacterial growth in the context of the whole *has* locus was affected when the charged residues of loop 4 of HasR were replaced by neutral and polar residues. The cells expressing HasR_L4_ and HasR_L4short_ mutants grew with a significantly longer lag phase (8 and 9 hours) compared to the wild type (3 hours) (Fig. 5B). After this extended lag phase, the growths reached a similar OD_600nm_ as in the presence of the HasR wild type. This suggests that L4 of HasR is involved in the initial steps of the mechanism. Consistent with this hypothesis, when the test is conducted overnight and in solid medium, the ability of L4 mutants to acquire heme was either not or only slightly modified (Fig. 5A, left panel).

The mutations of the L10 loop of HasR (HasR_L10short_ or HasR_L10_) almost completely abolished bacterial growth. This is consistent with the simulation showing L10 interacting with HasB when HasA and heme are present. The level of expression of HasR_L10short_ or HasR_L10_ mutants and their recognition at the surface of bacteria were similar to that of the wild type (Fig. S6A), showing that the mutant receptors are well folded and exposed at the bacterial surface, although their affinity for HasA seems to be affected (Fig. S6B). In solid phase, cells expressing these mutants were able to acquire heme but only at high heme concentration (Fig. 5A, right panel).

We also mutated the charged residues of HasB that were involved in salt bridges with the L4 and L10 loops of HasR in the simulations. The phenotypic assay with bacteria expressing HasB_K230AR231A_ variant (Fig. 5B) showed that the mutation of K_230_ and R_231_ of HasB (partner of HasR L4 in the simulations) abolished bacterial growth. The amount of this HasB variant in the cells was reasonable, albeit less than the wild type (Fig. S6C).

HasB residues R_223_ and E_224_ formed salt bridges with the L10 loop of HasR in the simulations. When these charged residues were replaced either with Ala or Asn, the amounts of HasB variants in the cells were highly affected (Fig. S6C). Given the minimum amount of HasB required for the activity of the Has system is not known, the lack of bacterial growth could be due either to a reduced amount of HasB mutants compared to the wild type or to the destabilization of the HasR-HasB interaction.

### Contribution of the HasR periplasmic loops to the HasR-HasB interaction

**Fig 6:**
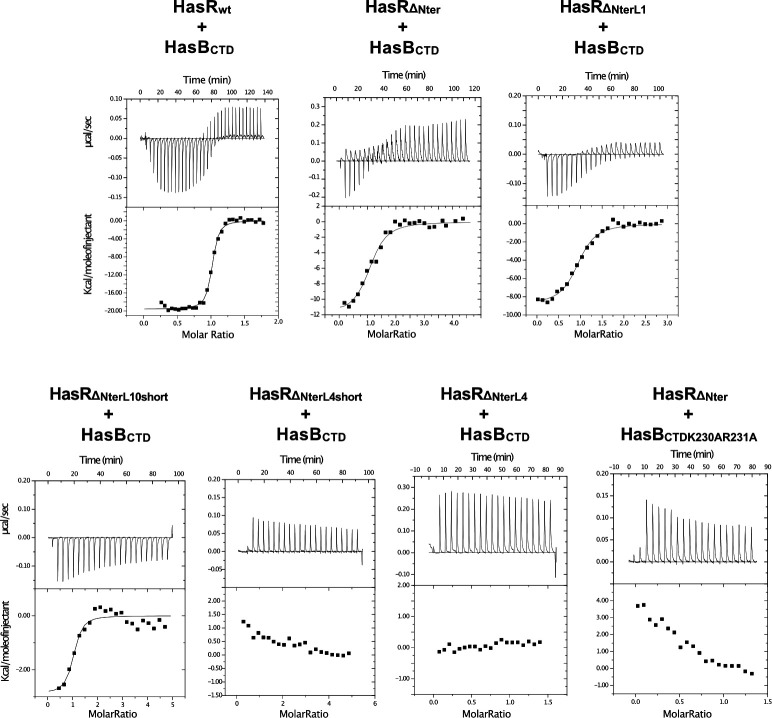
ITC analysis of the interaction of HasB_CTD_ and HasR variants. Representative experiments are shown. In each case, the heat signal is shown together with the binding isotherm derived from the signal. The titration data were analyzed by using the Microcal-ORIGIN software package. All protein samples were previously dialyzed in 20 mM sodium phosphate, pH 7, 0.08% Zwittergent 3–14. For all titrations, aliquots of HasB_CTD_ variants were injected into the ITC cell containing HasR proteins.

In a previous work, we studied the *in vitro* interaction of purified HasR and HasB_CTD_ by isothermal titration calorimetry (ITC) and quantified the thermodynamic parameters of the interaction (20). Here, to specifically measure the contribution of HasR periplasmic loops, we produced a HasR mutant, HasR_∆Nter_, by removing the SD, linker, and TonB-box. In the periplasmic face of this HasR mutant, only the loops are present and available to interact with HasB. As shown in Fig. 6; Table 2, HasR_ΔNter_ interacted with HasB_CTD_, although with significantly lower affinity (>30 times) and lower enthalpy of interaction (ΔH) compared to the wild type. Thus, the ITC experiment with the HasR_ΔNter_ mutant enabled us to isolate the contribution of HasR periplasmic loops in the HasR-HasB_CTD_ interaction.

**TABLE 2:** Binding energies from ITC analysis of the interaction between HasB_CTD_ and HasR variants^*a*^

Binding partners		N	*K*_a_ (M^−1^)	ΔH (kcal mol^−1^)
HasR_WT_	HasB_CTD_	1.03	7.50 (± 0.2) x 10^7^	−19.1 ± 0.24
HasR_ΔNter_	HasB_CTD_	1.10	2.30 (± 0.8) x 10^6^	−11.1 ± 0.8
HasR_ΔNterL1_	HasB_CTD_	1.10	3.03 (± 0.4) x 10^6^	−9.2 ± 0.19
HasR_ΔNterL10short_	HasB_CTD_	0.98	1.45 (± 1.0) x 10^8^	−2.9 ± 0.35

^*a*^ Stoichiometries (N), affinity constants (*K*_a_), and enthalpy changes (ΔH) of binding at 25°C were determined by fitting the binding isotherm from the ITC titration data by using the Microcal-ORIGIN software package to a model with one set of sites.

Next, to measure the individual contribution of L4 or L10, we made both the short and long versions of the mutations in these loops as in the *in vivo* experiments but with the HasR_ΔNter_ version. The effect of mutating L4 on the interaction with HasB was drastic. The ITC experiments testing the interaction of HasB_CTD_ with either HasR_ΔNterL4short_ or HasR_ΔNterL4_ displayed only small endothermic signals that corresponded to the heat of dilution (Fig. 6). Similar endothermic signals were observed when we mutated those residues of HasB that were observed to interact with HasR L4 in our MD simulations (HasR_ΔNter_- HasB_CTDK230AR231A_, Fig. 6, lower panel). The enthalpic signal reflects the contribution of polar interactions. The loss of the enthalpic signal observed with these mutants compared to the HasR_ΔNter_-HasB_CTD_ interaction (ΔH value, −11.1 kcal mol^−1^) is compatible with at least two salt bridges that could correspond to the contribution of the HasR L4-HasB K_230_R_231_ interaction.

The HasR_ΔNterL10short_ formed a complex with HasB_CTD_, although with lower affinity and a loss of polar interactions compared to HasR_ΔNter_ (ΔH, −2.9 *versus* −11.1 kcal mol^−1^) (Fig. 6; Table 2). The HasR_ΔNter-L10_ mutant was obtained in an aggregated form at the end of the purification and could not be tested. The aggregation due to the mutations can explain why HasR_L10_ was affected in its capacity to bind HasA at the cell surface, although the amount of mutant proteins in the total extract was comparable to that of the wild type (Fig. S6A and B).

Next, we wanted to test HasB_CTD_ variants in which R_223_ and E_224_, residues observed to interact with HasR L10 in our simulations, were mutated to Ala or Asn. Unfortunately, our attempts to obtain stable HasB_CTDR223AE224A_ or HasB_CTDR223NE224N_ variants were not successful. Both mutants oligomerized during the different steps of the purification and led to highly aggregated proteins that were eluted at the dead-volume of the size exclusion chromatography column. Similar destabilizing effect of these mutations was also observed *in vivo* in the context of the full-length HasB (Fig. S6C). The destabilizing effect of this mutation is most likely due to the disruption of critical interactions. Specifically, residues R_223_ and E_224_ in the central region of helix 219–231 form multiple salt bridges and polar interactions with other residues, which may be important in stabilizing the helix and the overall folding of HasB.

We also evaluated the contribution of HasR loop L1 to the HasR-HasB interaction. Mutation of this loop (P_232_GKE to ATSA) had no significant effect compared to the HasR_ΔNter_ (ΔH 9.2 ± 0.8 kcal mol ^−1^ for HasR_ΔNter-L1_-HasB_CTD_ versus 11.1 ± 0.8 kcal mol ^−1^ for HasR_ΔNter-L1_-HasB_CTD_) (Fig. 6; Table 2). This result aligns with the simulations showing that the interaction of HasB_CTD_ with HasR L1 mostly involves hydrophobic contacts.

The ITC experiments are consistent with the simulation data regarding the contributions of HasR periplasmic loops 1, 4, and 10 in the HasR-HasB interaction. Among the loops that were tested, L4 seems to show a higher contribution to the interaction, as also revealed by the simulations in the absence of heme and HasA. The contributions of polar and hydrophobic interactions of L10 or L1 in the HasR_ΔNter_-HasB_CTD_ were also experimentally demonstrated.

## DISCUSSION

Our study reveals key roles of specific periplasmic loops of a TBDT in its interaction with a TonB protein, and in its function in heme acquisition. Our simulations predict that the loops involved in the inter-protein interactions differ depending on the presence of the extracellular substrates, heme and HasA. This implies that the conformational and dynamics changes associated with substrate binding on the extracellular side of HasR may be transmitted through the barrel and plug to the periplasmic side of HasR and the region of interaction with HasB. It should be noted that only an integrative approach, combining *in silico* molecular modeling and dynamics in a membrane environment, along with *in vivo* and *in vitro* experiments, as demonstrated here, can provide detailed atomistic-level insights into molecular interactions of such a system between two membranes.

In the absence of extracellular substrates, the interaction of HasR L1 and HasB occurs mainly *via* hydrophobic contacts. Once these contacts are formed, the HasR linker and HasB move with respect to each other (mostly through HasB rotation) enabling HasB to make contact with the HasR L4 (Fig. 1; Movie S1). The HasR-HasB interaction is then stabilized through several salt bridges between L4 and HasB K_230_R_231_ residues (Fig. 4A).

*In vivo* when the charged residues in L4 (HasR_L4short_ and HasR_L4_ variants) or in its partner on HasB (HasB_K230AR231A_ variant) were removed, the bacterial growth was significantly affected (Fig. 5B) with a long lag phase suggesting the key role of the L4-HasB interaction at the early stage of the HasR-HasB interaction. The ITC experiments with different mutants showed also that HasR L4 and its partners on HasB are required for HasR-HasB complex formation and stability (Fig. 6).

In the simulations, when the extracellular substrates are present, although L4 and L1 of HasR rapidly make contact with HasB, the interactions with L4 are disrupted. Instead, residues of L10 form polar and ionic interactions with HasB (Fig. 4C). We validated the key role of the charged residues of HasR L10 in the interaction with HasB through *in vitro* and *in vivo* functional assays (Fig. 5 and 6). The *in vivo* data showed that the mutation of HasR L10 or L4 modified bacterial growth. While the role of L4 seems crucial in the early stages of heme acquisition, mutations of L10 significantly affect bacterial growth. This suggests that the HasR L10-HasB interaction may play a crucial role in heme internalization and the ejection of HasA, two steps that require energy (13).

Structural data showed that the TonB-TBDT interaction involves a salt bridge between the TonB-box of the transporter and TonB, stabilizing an inter-protein β-sheet (5, 7, 8, 19). Both salt bridge and the inter-protein β-sheet were shown to be important for the energy transfer from the TonB protein to the transporter (30, 31). In all the simulations presented here, the salt bridge between the HasR TonB-box and HasB and the inter-protein β-sheet are consistently formed, although they are often observed to disrupt and reform.

If we compare the HasB-HasR interaction network with the sequence and the structures of other available TBDT-TonB complexes (Protein Data Bank entries 2GSK, 2GRX, and 6I97), the residues of HasR L1 and L4—notably the charged residues of L4—are well conserved in FoxA, BtuB, and FhuA, although the loops are slightly shorter in these proteins compared to those in HasR. HasR L10 appears to be less conserved (Fig. S7). The HasB residues that contact L1, L4, and L10 in our simulations are also well conserved and exposed at the surface of *E. coli* and *P. aeruginosa* TonB (Fig. S7). However, no simultaneous interactions among TBDT L1, L4, and L10 with TonB are observed in these complex structures possibly due to the presence of only the globular C-terminal part of TonB. In our study, a full-length model of HasB is inserted into the IM. Its dynamics in the context of the envelope would enable its simultaneous interaction with HasR L1 and L4 or L10. Therefore, we speculate that the HasR-HasB interaction mode revealed in this study can occur in other TBDT-TonB complexes.

The energizing mechanism used by Gram-negative bacteria to internalize scarce nutrients is still not well deciphered. It is assumed that energized TonB applies a mechanical force to remodel the TBDT plug and open a channel through the barrel, allowing substrate entry. How TonB applies this force is not yet understood. Recently, we obtained structural and mechanistic information on the conformational switch of ExbD, its interaction with HasB, and the role of peptidoglycan in the energy transfer mechanism (32). These data along with EPR (33), AFM (31), and steered molecular dynamics data on homologous TBDT-TonB systems (30) suggest a rotational mechanism similar to that of the Mot flagellar system (10), accompanied by a pulling movement of TonB. During our simulations, we did not observe any pulling movement of HasB (Movie S1), as the HasB_CTD_-HasR barrel distance remains stable (4–5 nm in all the simulations). However, the simulations are performed in the absence of other partners in the IM (ExbB and ExbD) (4) and any source of energy. Further studies including ExbB, ExbD, and peptidoglycan will thus be required to fully understand the dynamic mechanism of energy transfer between the two bacterial membranes. A more in-depth understanding of this mechanism may facilitate the development of new antibacterial strategies against pathogenic Gram-negative bacteria.

## MATERIALS AND METHODS

### Positioning the protein complex in the membranes

The CHARMM-GUI webserver (25, 34) was used to assemble the lipid bilayers, both as squares with a side length of 16.5 nm. The IM was made up of two leaflets of 1-pamitoyl-2-oleoyl-sn-glycero-3-phosphoethanolamine (POPE). The OM similarly had a lower leaflet of POPE but an upper leaflet of 1,2-dilauroyl-sn-glycero-3-phosphocholine (DLPC) as the tail lengths of DLPC are similar to those of lipopolysaccharide (which is found in the outer leaflet *in vivo* but requires long simulations to achieve convergence and thus was not used here, as our interest was in the protein complex rather than specific details of protein-outer membrane interactions) (26). The two membranes were subjected to a 5,000-step energy minimization using the steepest descents algorithm.

To align the HasR barrel in the OM, we took coordinates from the MemProtMD database (35) and used them for alignment to ensure the correct position of lipid headgroups relative to the protein. The location of the HasB helix in the IM was estimated by using the location of the tryptophan residues, given that there was no structural information available for this section (36).

Simulations involving HasR and HasB were set up as follows: once the proteins had been inserted into the membranes, any overlapping lipids were removed prior to energy minimization. The CHARMM-GUI webserver was used to generate the necessary topology and parameter files for all models and structures reported in this study.

### Molecular dynamics simulations

The systems were solvated with the TIP3P water model. K^+^ and Cl^-^ ions were added to achieve an overall concentration of 150 mM in charge neutral systems. The systems were then subjected to a short energy minimization of 5,000 steps, again by using the steepest descent algorithm, to remove any steric clashes, followed by an equilibration protocol as shown in Table S1. This protocol, with slow relaxing of the restraints on the protein, was necessary to allow the lipids to equilibrate around the proteins.

All simulations were carried out with the GROMACS 2019.6 (37) version (www.gromacs.org) and CHARMM36 (38) forcefield. The long-range electrostatic interactions were treated by using the Particle Mesh Ewald (PME) (39) method, whereas the nonbonded interactions and the short-range electrostatics were cut off at 1.2 nm, applied by using the potential shift Verlet cut off scheme. The temperature was maintained at 303.15 K by using the Nosé-Hoover thermostat (40, 41) with a 5 ps coupling constant. To maintain a constant pressure at 1 atm, we applied the Parrinello-Rahman barostat (42) in a semi-isotropic fashion, again with a coupling constant of 5 ps. The LINCS (43) algorithm was applied to all atoms to allow the time step of 2 fs. Table 1 shows the summary of production runs, all of which were run in the NPT ensemble.

### Strains and plasmids

The *E. coli* strains and plasmids used in this study are listed in Table S2 and described in the Supplementary Information.

### Protein production and purification

HasR wild type and mutants were expressed in M9 16L fermentor and purified as previously described (13, 16). Briefly, after the extraction of proteins from the membrane with Zwittergent 3–14, the purification was performed in two steps: (i) an anion-exchange chromatography with a Q-HP 16/10 column (*Cytiva*) equilibrated in 50 mM Tris-HCl and 0.04% Zwittergent 3–14. The bound proteins were eluted in the same buffer supplemented with 1 M NaCl and with a linear gradient. (ii) A size exclusion chromatography with a Hi-Prep 26/60 Sephacryl S-300 column (*Cytiva*) equilibrated in 20 mM sodium phosphate, pH 7, and 0.04% Zwittergent 3–14.

HasB_CTD_ wild type and mutants were expressed and purified as described in our previous work (20). Briefly, the cytoplasmic fraction was purified in two steps: (i) a cation exchange chromatography with a SP-HP 16/10 column (*Cytiva*) equilibrated in 50 mM Tris-HCl and 100 mM NaCl, pH 8.5. The bound proteins were eluted in the same buffer but with 1 M NaCl and with a linear gradient. (ii) A size exclusion chromatography with a HiLoad Superdex 75 (16/600) (*Cytiva*) equilibrated in 50 mM sodium phosphate and 50 mM NaCl, pH 7. All the purification steps were performed at 4°C or 12°C in the presence of a protease inhibitor cocktail (*cOmplet, Roche*). Protein concentrations were estimated using UV absorbance and the following calculated or determined molar absorption coefficients: 140,000 M^−1^ cm^−1^ at 280 nm for HasR wild type and mutants (13) and 10,000 M^−1^ cm^−1^ at 280 nm for HasB_CTD_ and mutants (20).

### Protein expression level and activity test at the cell surface

Protein expression levels were measured in two different contexts for HasR, HasB, and their derivatives. Regarding the expression of HasR and its derivatives, C600*∆hemA* strain transformed with various pFR2 recombinant plasmids and was grown up to an OD_600nm_ of 0.2–0.3 in LB medium at 37°C, and protein expression was induced by adding 40 µg/mL arabinose. After 2 hours, the culture was TCA-precipitated, and the equivalent of 0.15 OD_600nm_ was loaded on SDS-PAGE and transferred on nitrocellulose. HasR was detected with anti-HasR antibodies. Alternatively, on the same culture, HasR at the outer membrane was tested on whole cells by dot-blot with anti-HasR antibodies. We also evaluated its ability to bind HasA by dot-blot after incubation of the dot-blot with various concentrations of HasA and detection of HasA bound to the cell surface by HasR using anti-HasA antibodies.

For HasB and its derivatives, C600*∆exbBD* strain was transformed with pBAD24HasB or its derivatives together with pBAD33ExbBDSm, as HasB expression is stabilized by the co-expression of ExbB and ExbD. Protein expression was induced by arabinose for 2 hours at 37°C. The culture was TCA-precipitated, and HasB was detected in whole cells by immunodetection using anti-HasB antibodies after running a whole cell sample (equivalent of 0.15 OD_600nm_) on SDS-PAGE.

### Bacterial growth and phenotypic tests

Bacterial phenotypic tests were carried out both on solid and in liquid media. On solid medium, C600*∆hemA E. coli* strain expressing only HasR (from pFR2 plasmid or its derivatives) was grown up to an OD_600nm_ of 1, and 100 µL of this culture was mixed with 3.5 mL of soft agar and spread on the top of a Petri dish. Holes were punched in both layers and filled with heme-BSA at 0.4, 2, or 10 µM. The plates contained 40 µg/mL arabinose, plus or minus 150 µM dipyridyl to induce TonB complex expression.

To test in liquid medium, we used *E. coli* strain C600*∆hemA∆exbBD* transformed with two plasmids. First, one pAMHasISRADEB encoding the whole Has locus (*HasISRADEB*) required for HasA secretion (*HasA*, *HasD*, and *HasE*), heme internalization (*HasR* and *HasB*) under Fur regulation (induced by iron starvation conditions), as well as the transcription regulation of the system (*hasI* and *hasS*) regulated with the presence of both HasA and heme on their respective binding sites on the HasR receptor (15). A second vector pBAD24exbBDsm encoding ExbB and ExbD from *S. marcescens* under the control of the *araBAD* promoter was used. A few colonies of the recombinant strain were inoculated in 4 mL of LB medium at 37°C with 100 µM dipyridyl (an iron chelator), 4 µg/mL arabinose (to induce expression of ExbB and ExbD), and the corresponding antibiotics. Once the culture reached an OD_600nm_ of approximately 1.2–1.5, it was diluted at 0.001 OD_600nm_ and inoculated in 48-well Greiner plates, using the same medium supplemented with 1 µM heme-BSA, as the heme source. Each well contained 300 µL of growth medium. Duplicates or triplicates of each strain were made, and the plates were incubated at 37°C with vigorous shaking (500 rpm) in a *Clariostar Plus Microplate* reader. The OD_600nm_ was recorded every 15 minutes for 30 hours. All experiments were performed in triplicate. Strains and plasmids used in this study are described in the Supplementary Information and listed in Table S2.

### Isothermal titration calorimetry (ITC)

ITC experiments were performed at 25°C by using a MicroCal VP titration calorimeter (MicroCal, GE Healthcare) under the same conditions as described previously (20). The buffer was 20 mM sodium phosphate, pH 7, and 0.08% Zwittergent 3–14 for all titrations. Purified protein samples were dialyzed in this buffer before the experiments. Consecutive aliquots of 5–10 μL of HasB_CTD_ at 80–200 μM were added into the ITC cell containing HasR wild type or mutant proteins at 4–15 μM. The heat of dilution was determined by injecting the same aliquots of HasB_CTD_ variants into the cell either after the saturation or containing the buffer. The value obtained was subtracted from the heat of reaction to give the effective heat of binding. The resulting titration data were analyzed by using the Microcal-ORIGIN software package. The molar binding stoichiometry (N), association constant (*K*_a_), and enthalpy changes (ΔH) of binding were determined by fitting the binding isotherm to a model with one set of sites. All experiments were done in duplicate and with different batches.

## SUPPLEMENTAL MATERIAL

Supplemental Material mbio.01781-24-s0001.docx Supplemental methods, Fig. S1 to S7, Tables S1 and S2, and legend for Movie S1.

Movie S1 mbio.01781-24-s0002.gif The video is taken from the first 120 ns of one of the HasR-HasB system simulations.

ASM does not own the copyrights to Supplemental Material that may be linked to, or accessed through, an article. The authors have granted ASM a non-exclusive, world-wide license to publish the Supplemental Material files. Please contact the corresponding author directly for reuse.
