# Supplementary material for: Dynamic interplay between a TonB-dependent heme transporter and a TonB protein in a membrane environment
Source: mBio. 2024 Oct 30;15(12):e01781-24. doi: 10.1128/mbio.01781-24 (PMC11633176; doi:10.1128/mbio.01781-24)
Supplement: Supplemental Material — Supplemental methods, Fig. S1 to S7, Tables S1 and S2, and legend for Movie S1. [file mbio.01781-24-s0001.docx]

**Supplementary Information for**

**Dynamic interplay between a TonB dependent heme transporter and a TonB protein in a membrane environment**

Kamolrat Somboon^1^, Oliver Melling^1^, Maylis Lejeune^2^, Glaucia M.S. Pinheiro^5^, Annick Paquelin^3^, Benjamin Bardiaux^2^, Michael Nilges^2^, Philippe Delepelaire^3^, Syma Khalid^1,4*^, Nadia Izadi-Pruneyre^2*^

*Corresponding authors: Syma Khalid & Nadia Izadi-Pruneyre

**Email:**  [syma.khalid@bioch.ox.ac.uk](mailto:syma.khalid@bioch.ox.ac.uk), [nadia.izadi@pasteur.fr](mailto:nadia.izadi@pasteur.fr)

**This PDF file includes:**

Supplementary text

Figures S1 to S7

Table S1-S2

Legend of Movie S1

SI References

Supplementary Text

**Methods**

**Construction of the protein complex model**

The HasR structure used as the starting point was a model that combined the X-ray structure of the HasR barrel and plug (PDB entry 3CSL)(1), the NMR structure of HasR_SD_ (PDB entry 2M5J)(2), SAXS data and the EM map of HasR (EMD-3978), as detailed by Wojtowicz *et al.*(2) In a previous work(3), a 3D model had been generated between the TonB-box of HasR and the C-terminal domain of HasB (PDB 2M2K) based on NMR experimental data (chemical shift perturbation and distance restraints) by using the docking program Haddock (4). This structure included a 21-residue peptide of the TonB-box of HasR, from G92 to A112, along with the C-terminal domain of HasB, N167 to F263 inclusive. This model was used to replace the equivalent region in the HasR starting structure by using the PyMol software package (PyMol Molecular Graphics System, Version 1.2r3pre, Schrödinger, LLC.) We note here that a more recent structure of HasR (PDB 5C58) has been determined (5), but due to the missing extracellular loops this was not used in the present study. For the simulations with the holoHasR (HasA-heme-HasR), HasA and heme were positioned using Visual Molecular Dynamics (VMD)(6) according to the 3CSL PDB structure (1).

The HasR_L4_ variant was made by removing the L4 (P_392_EDVDWLD) residues of HasR, before using Modeller to add the ANATSANA sequence. The H++ web-server (7–9) was then used to add hydrogen atoms to the new residues.

The HasB structure was modelled using the NMR structure of its C-terminal domain (residues 133 to 263, PDB 2M2K). The PEP-Fold web-server (10–15) was used to generate a model of the HasB transmembrane helix. The Modeller software (<https://salilab.org/modeller>)(16, 17) was used to generate the periplasmic proline-rich domain (residues 37 to 132) that is disordered and located between the transmembrane helix and the C-terminal part, and ModLoop(18), a module of Modeller, was used to model the missing peptide bonds when connecting the various portions of the proteins.

**Strains and plasmids**

The *E. coli* strains and plasmids used in this study are listed in Table S2. The plasmids for the expression of HasR mutants (pFR2 derivatives HasR_L1_, HasR_L3_, HasR_L4short_ and HasR_L10short_) were prepared as follows: pFR2 plasmid (lab collection) was amplified by PCR with two couples of oligonucleotides (delP3-5 and delP3-3, on the one hand, and delP4-5 and delP4-5, on the other that contained a *Spe*I site at their 5’ extremity). The PCR product was digested with *Spe*I, self-ligated and transformed into *E. coli* XL1-Blue. Recombinant plasmids were isolated and correct ones selected after sequencing, and the insert bearing the mutation reinserted in an otherwise wild-type pFR2 plasmid. The pFR2HasR_L1_, pFR2 HasR_L4short_ and pFR2HasR_L10short_ plasmids were thus obtained, replacing for HasR_L1_ A_238_PGKEL_243_ by AATSAL, for HasR_L4short_ A_391_PEDVDWLDF_400_ by AATSAF and for HasR_L10short_ G_773_RAFDRKLDA_782_ by GATSAA. The mutants were also cloned into the pAMHasISRADEB context, by exchanging the *Fse*I-*Bsi*WI fragments between pFR2 mutants and pAMHasISRADEB. The same procedure was applied for mutants pFR2HasR_L4_ and pFR2HasR_L10_, with the respective oligonucleotide couples 5'loop4pol/3’loop4pol and 5'loop10pol/3’loop10pol; the pFR2HasR_L4_ and p pFR2HasR_L10_ where thus obtained, replacing for HasR_L4_ A_391_PEDVDWLDF_400_ by AANATSANAF and for HasR_L10_ G_773_RAFDRKLDA_782_ by GANATSANAA.

The HasB mutants were first built into pHasBAD24 plasmids, with the mutagenic oligonucleotide couples HASBKRAA2/HASBKRAA2r, HasBRE223-224AA/HasBRE223-224Aar and HasB223N/HasB224N. Likewise the mutants were reintroduced into pAMHasISRADEB, by replacing the *Bsu*36I-*Hin*dIII fragment from pAMHasISRADEB by the one from pHasBAD24. The plasmids for the expression of HasB_CTD_ wild type (19) and mutants were pBAD derivatives, and were prepared by *Proteogenix* *(Schiltigheim, France)*.

**Protein production and purification**

Now presented in the main text.

Supplementary figures and tables


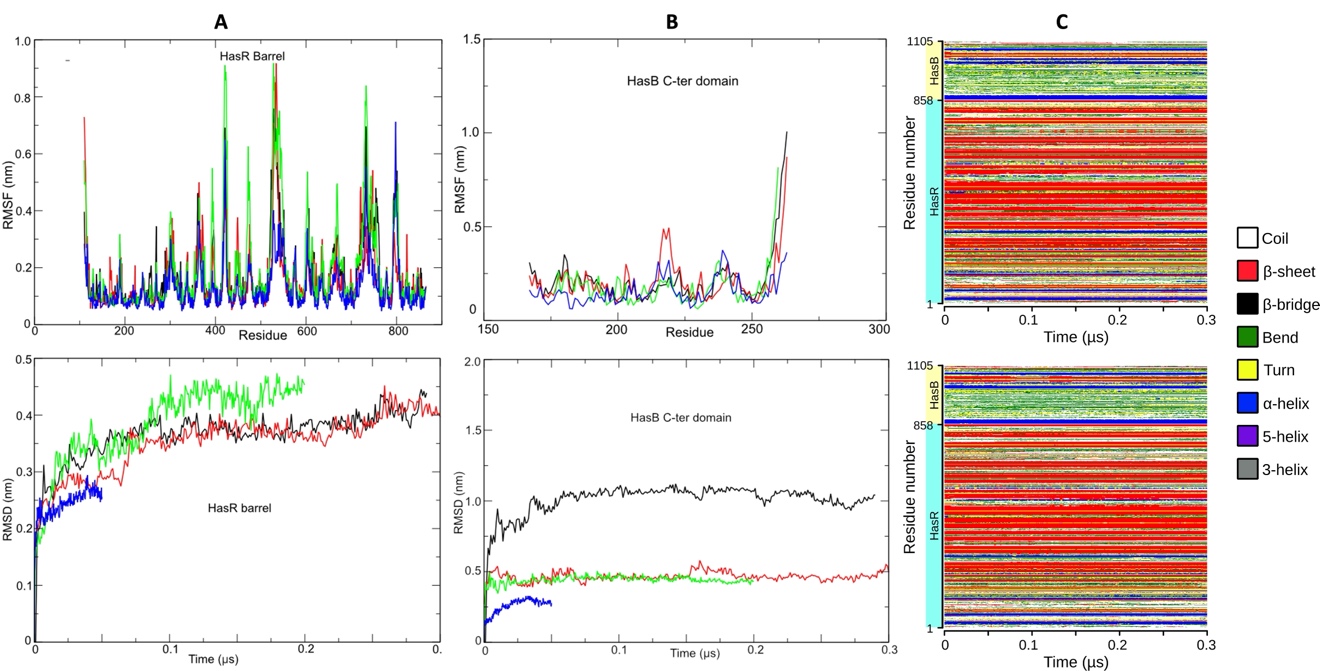


**Figure S1. Secondary structure and structural integrity of the HasR-HasB complex. A**: RMSF (top) and RMSD (bottom) plots of the HasR barrel. **B**: RMSF (top) and RMSD (bottom) plots of the HasB_CTD_. The curves are colored as follows, HasR-HasB (black), holoHasR-HasB (red), HasR_L4_-HasB (green), HasR-HasBwide (blue). **C**: Secondary structure vs time analyses for HasR-HasB (top) and holoHasR-HasB (bottom).

**
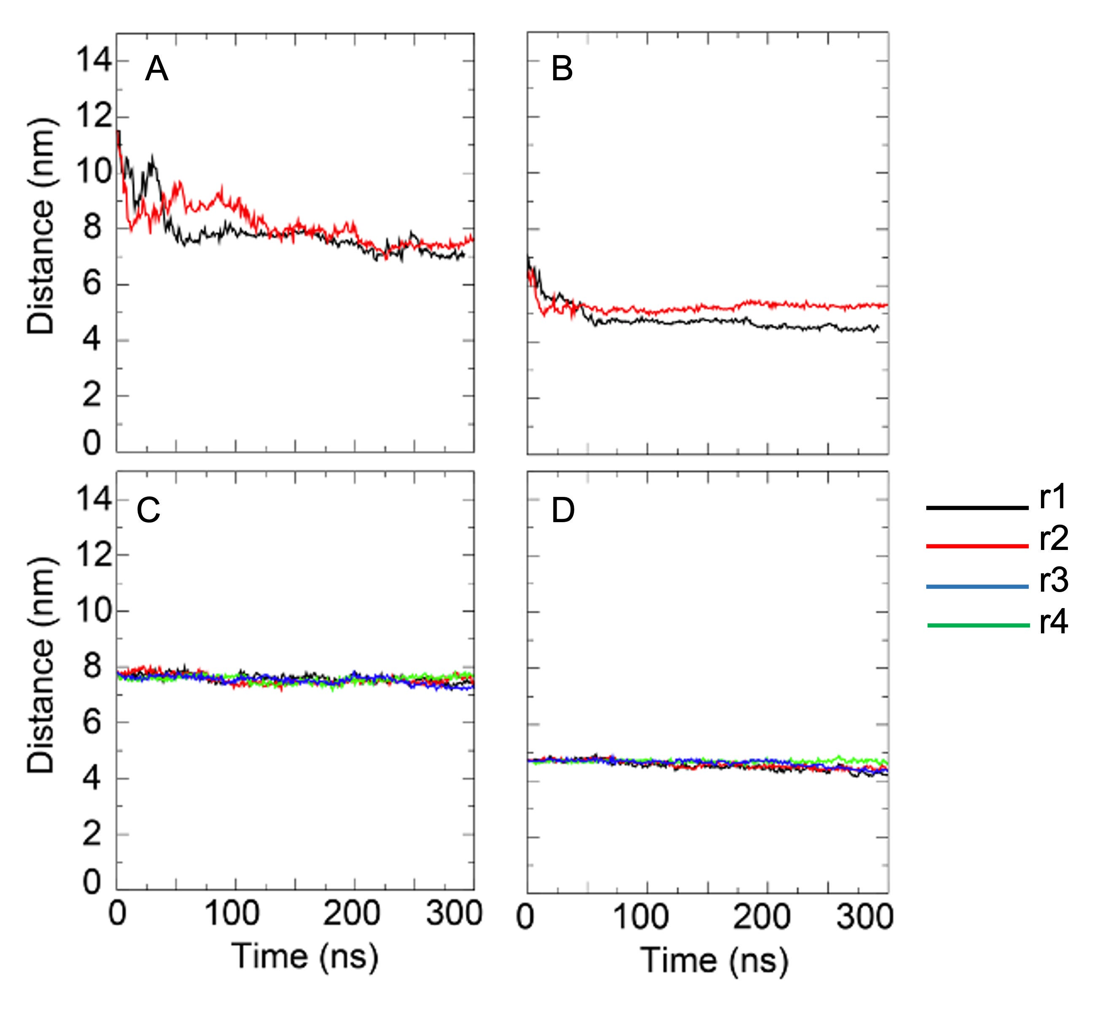
**

**Figure S2. Intra- and inter-protein domain distances.** Panels **A** and **C** show the minimum distance between the HasR barrel and the HasR_SD_ as a function of time in the HasR-HasB and int-HasR-HasB simulations respectively. Panels **B** and **D** show the minimum distance between the HasR barrel and the HasB_CTD_ as a function of time in the HasR-HasB and int-HasR-HasB simulations respectively. In each case the center of mass of the protein domain is used for the measurements. Different simulations from r1 to r4 are presented.

**Figure S3. Inter-protein interaction modes in the two HasR_L4_-HasB simulations. A.** Model of HasR_L4_ mutant and HasB at the beginning of the simulations. HasR is in cyan, HasB in pink, The TonB box, L1, L4 and L10 are in dark blue, yellow, red and green respectively. The HasB TM is not shown for clarity. **B.** Snapshots from the end of the two HasR_L4_-HasB simulations showing different binding mode and HasB positioning.


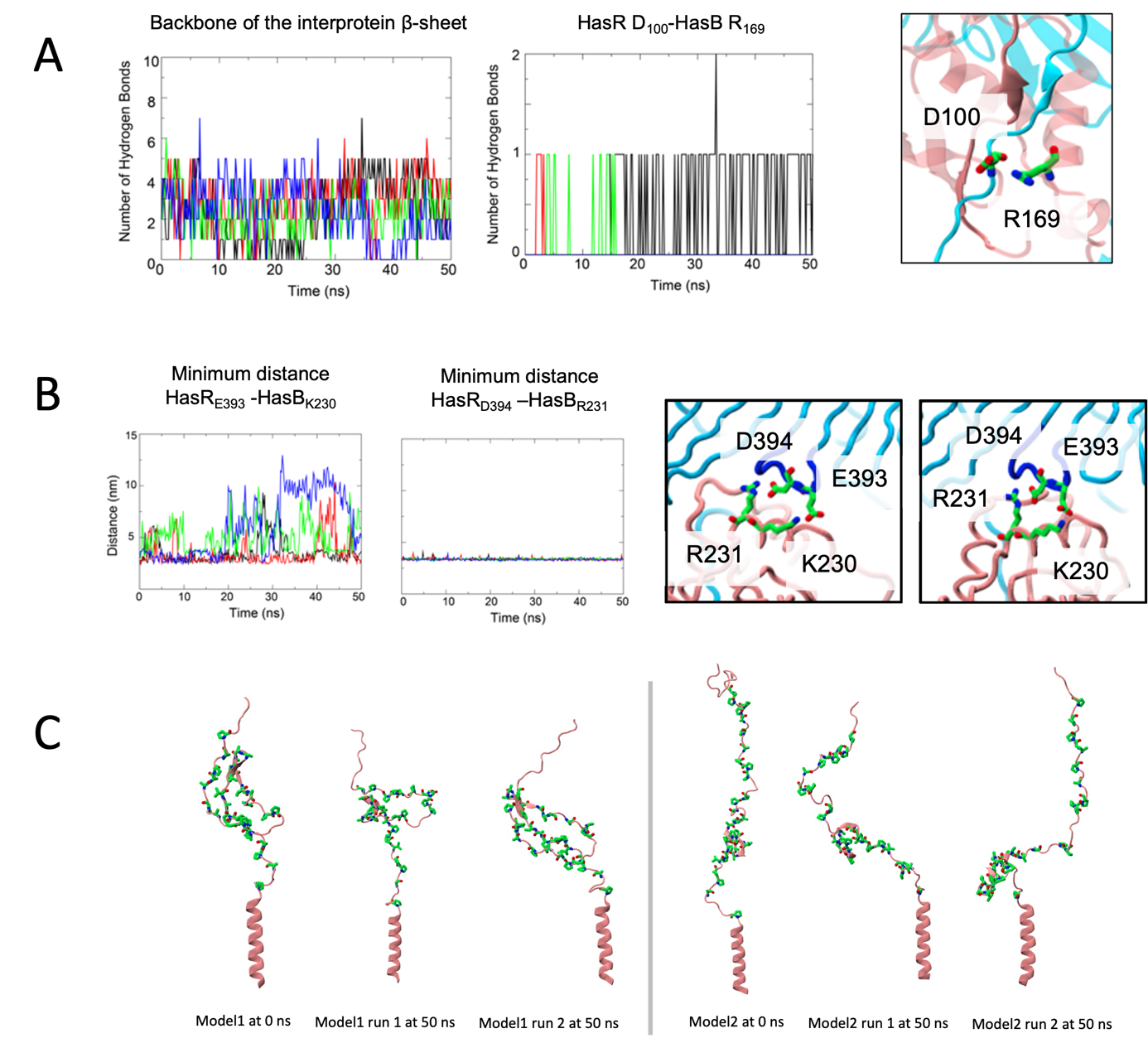


**Figure S4*.* Analysis of the HasR-HasB interaction in the simulations with the wide periplasm (at 23 nm). A:** Hydrogen bond analysis for the interprotein β-sheet (HasR_93-111_-HasB_246-258_) and the HasB-HasR TonB box interaction (HasR D_100_-HasB R_169_). Each simulation is represented in a different color. A representative snapshot at 20ns showing the HasR D_100_-HasB R_169_ interaction is presented on the right. **B:** Minimum distance between HasR L4 and HasB (left and central panel) with snapshots at 20 ns from different runs (right panel) are presented. **C:** Hydrophobic clustering in the proline-rich region of HasB during the simulations. The hydrophobic residues Val, Ala, Ile and Pro from the stretch 37-90 of HasB are shown in stick. In all structures, HasR is in cyan, HasB in pink and some important residues are presented as stick and labelled. All the analysis were performed by using VMD software.

**
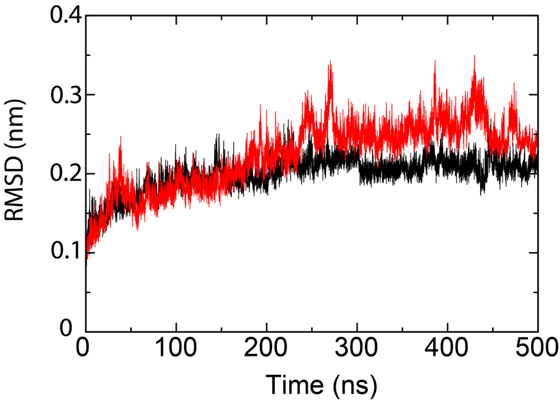
**

Run 1

Run 2

**Figure S5.** Simulations of HasR in an outer membrane model containing lipopolysaccharide (LPS) at the Ra-LPS level (*i.e.* without the O-antigen group). RMSD of the C atoms during the two x 500 ns simulations.

**Figure S6. Amount of HasB and HasR variants in bacterial cells and activity tests**. **A:** Expression level of HasR and its derivatives analysed in western blot with anti-HasR antibody. Total extracts from 0.15 OD600_nm_ of strain C600*∆hemA* harbouring pBAD derivative plasmids, pFR2 for the expression of HasR_WT_ or its variants were precipitated with TCA, run on SDS-PAGE and transferred on nitrocellulose membrane. Standard molecular weight (MW) markers are run on the left. **B:** Whole cells from the same cultures as in A were analysed by dot-blot with anti-HasR antibodies (left panel) and for their ability to bind HasA (right panel) after incubation of the dot-blot with various concentrations of HasA (1, 10 and 100 nM) and detection of HasA bound at the cell surface with anti-HasA antibody. All immunoblots were revealed with goat anti-rabbit IgG coupled to alkaline phosphatase. **C:** Expression level of HasB and its derivatives analysed in western blot with anti-HasB antibody. Equivalent 0.15 OD_600nm_ of a total extract of strain C600*∆exbBD* complemented with pBAD33ExbBDSm and pBAD24 encoding HasB wild type or its derivatives, was precipitated with TCA, run on SDS-PAGE and transferred to the nitrocellulose membrane. Standard molecular weight (MW) markers are run on the left.


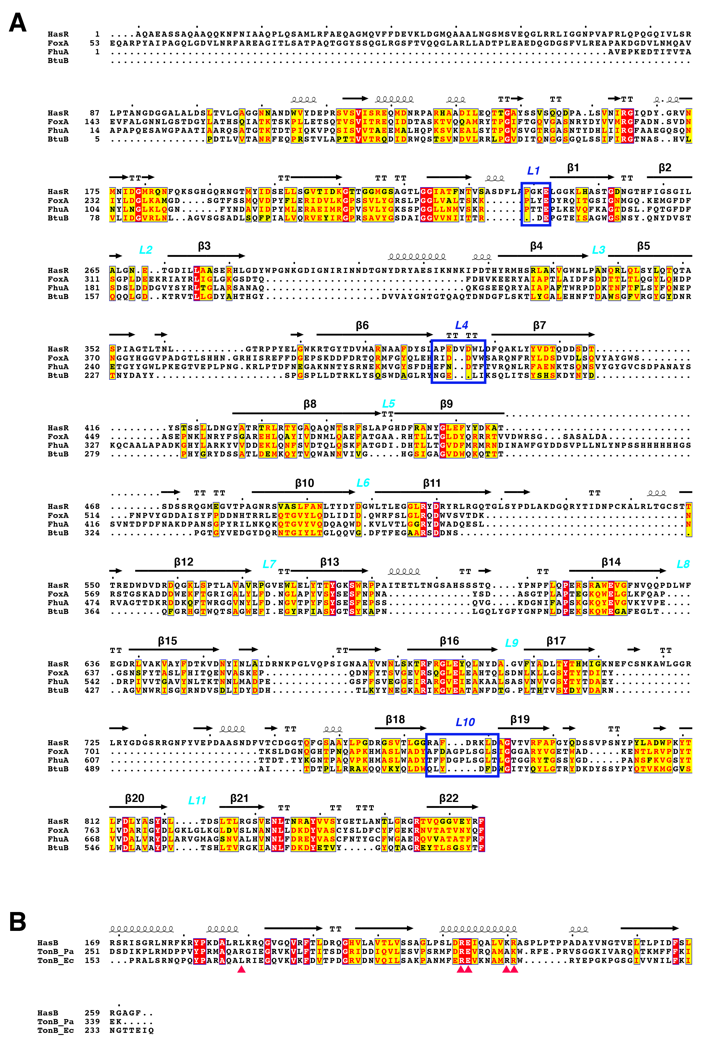


***Figure S7. Structural alignment of TBDT and TonB sequences.*** ***A****: Structural alignment of TBDT HasR (PDB 3CSL), FoxA (PDB 6I97), FhuA (PDB 2GRX) and BtuB (PDB 2GSK) sequences. Positions of the 22 β-strands of the β-barrel are labelled along with the 11 periplasmic loops (blue). Blue boxes highlight the corresponding sequences for the periplasmic loops L1, L4 and L10.* ***B****: Alignment of HasB_CTD_ (PDB 2M2K) and TonB_CTD_ from Pseudomonas aeruginosa (PDB 6I97) and Escherichia coli (PDB 2GRX and 2GSK). Residues of HasB_CTD_ involved in HasR interaction from MD simulations are marked (red triangle).*

Table S1. Equilibration protocol of the simulations

N: constant number; P: pressure; T : temperature ; V : volume

| Step | Time Step (fs) | Total Time (ns) | Protein Backbone Restraints (kJ mol^-1^ nm^-2^) | Ensemble |
| --- | --- | --- | --- | --- |
| 1 | 1 | 0.25 | 2000 | NPT |
| 2 | 1 | 0.5 | 2000 | NPT |
| 3 | 1 | 0.75 | 2000 | NVT |
| 4 | 1 | 1.0 | 2000 | NVT |
| 5 | 1 | 1.5 | 1500 | NPT |
| 6 | 1 | 2.0 | 1500 | NVT |
| 7 | 1 | 2.5 | 1000 | NPT |
| 8 | 1 | 3.0 | 1000 | NVT |
| 9 | 2 | 4.0 | 500 | NPT |
| 10 | 2 | 5.0 | 500 | NVT |
| 11 | 2 | 6.5 | 250 | NPT |
| 12 | 2 | 8.0 | 250 | NVT |
| 13 | 2 | 10.0 | 100 | NPT |
| 14 | 2 | 12.0 | 100 | NVT |

Table S2. Strains, plasmids and oligonucleotides

**Strains:**

*Escherichia coli* K12 strains

XL1-Blue: cloning strain Lab collection

*C600∆tonB∆exbBD∆hemA::Km Lab* collection

*C600∆hemA::Km* Lab collection

JP313 Lab collection

Popc4420 Lab collection

**Plasmids:**

pBAD24 Control

pFR2 HasR wild-type (Izadi-Pruneyre *et al.,* 2006)

pFR2L4short HasR_L4short_ (This work)

pFR2L4 HasR_L4_(This work)

pFR2L10short HasR_L10sort_ (This work)

pFR2L10 HasR_L10_ (This work)

pBAD24HasB HasB wild-type cloned into pBAD24 (Lab collection)

pBAD24HasB_KR230-1AA_ HasB_K230AR231A_ (This work)

pBAD24HasB_RE223-4NN_ HasB_R223NE224N_ (This work)

pBAD24HasB _RE223-4AA_ HasB_R223AE224A_ (This work)

pBAD33ExbBD_Sm_ ExbBDSm (Biou *et al*., 2022)

pBAD33ExbBD_Sm_ ExbBDSm (Biou *et al*., 2022)

pAMhasISRADEB HasI, HasS, HasR, HasA, HasD, HasE, HasB (Biou *et al*., 2022)

pAMhasISR_L4short_ADEB HasI, HasS, HasR_L4short_, HasA, HasD, HasE, HasB (This work)

pAMhasISR_L10short_ADEB HasI, HasS, HasR_L10short_, HasA, HasD, HasE, HasB (This work)

pAMhasISR_L4_ADEB HasI, HasS, HasR_L4_, HasA, HasD, HasE, HasB (This work)

pAMhasISR_L10_ADEB HasI, HasS, HasR_L10_,HasA, HasD, HasE, HasB (This work)

pAMhasISRADEB_KR230-1AA_ HasI, HasS, HasR, HasA, HasD, HasE,HasB_K230R231A_ (This work)

pAMhasISRADEB_RE223-4NN_ HasI, HasS, HasR, HasA, HasD, HasE, HasB_R223NE224N_ (This work)

pAMhasISRADEB_RE223-4AA_ HasI, HasS, HasR, HasA, HasD, HasE, HasB_R223AE224A_ (This work)

pBAD24HasR∆Nter HasR∆Nter (Wojtowicz *et al.,* 2016)

pBADHasB_133_ HasB_CTD_ (Lefevre *et al*., 2008)

pBADHasB_133K230AR231A_ HasB_CTDK230AR231A_ (*Proteogenix*)

pBADHasB_133R223NE224N_ HasB_CTDR223NE224N_ (*Proteogenix*)

pBADHasB_133R223AE224A_ HasB_CTDR223AE224A_ (*Proteogenix*)

**Oligonucleotides**

HASBKRAA2: CAGGCGCTGGTCGCAGCCGCGTCGCCGCTG

HASBKRAA2r: CAGCGGCGACGCGGCTGCGACCAGCGCCTG

HasBRE223-224AA: CCGTCGCTGGATGCTGCAATCCAGGCGCTG

HasBRE223-224AAr: CAGCGCCTGGATTGCAGCATCCAGCGACGG

DelP1-5: CC**ACTAGT**AGCCGCCAGGAAATCGCTCGCGCT

DelP1-3: AA**ACTAGT**GCTCTGGGCGGCAAGCTGCACGCC

DelP3-5: CC**ACTAGT**AGCCAGATTCCAGCCCACCTTGGC

DelP3-3: AA**ACTAGT**GCTCAGCGCCTGCAGCTGAGTTAT

DelP4-5: CC**ACTAGT**AGCCAGGCTGTAGTCGAACGCCGC

DelP4-3: AA**ACTAGT**GCTTTTCAGGCCAAGCTGTATTAC

DelP10-5: CC**ACTAGT**AGCCCCGCCCAGCGTCACCGAACC

DelP10-3: AA**ACTAGT**GCTGCCGGGGTGACCGTACGCTTT

3'loop4NHasR: [PHO]AACTGGCTCAATTTTCAGGCCAAGCTGTAT

5'loop4NHasR: [PHO]GACATTGTTCGGCGCCAGGCTGTAGTCGAA

3'loop10NHasR: [PHO]AACAATCTGAACGCCGGGGTGACCGTACGC

5'loop10NHasR: [PHO]ATTGAAGGCATTCCCGCCCAGCGTCACCGA

5'loop4pol: GC**ACTAGT**CGCGTTCGCCGCCAGGCTGTAGTCGAACGC

3'loop4pol: CG**ACTAGT**GCGAACGCGTTTCAGGCCAAGCTGTATTAC

5'loop10pol: GC**ACTAGT**CGCGTTCGCCCCGCCCAGCGTCACCGAACC

3'loop10pol: CG**ACTAGT**GCGAACGCGGCCGGGGTGACCGTACGCTTT

***Movie S1: HasR-HasB interaction.*** *The video is taken from the first 120 ns of one of the HasR-HasB system simulations (r1). The HasR loop L1, L4 and L10 are colored in red, blue, and purple, respectively.*

**SI References**

1. S. Krieg, *et al.*, Heme uptake across the outer membrane as revealed by crystal structures of the receptor-hemophore complex. *Proc Natl Acad Sci U S A* **106**, 1045–1050 (2009).

2. H. Wojtowicz, *et al.*, Structural basis of the signalling through a bacterial membrane receptor HasR deciphered by an integrative approach. *Biochem J* **473**, 2239–2248 (2016).

3. G. C. de Amorim, *et al.*, The structure of HasB reveals a new class of TonB protein fold. *PLoS One* **8**, e58964 (2013).

4. G. C. P. van Zundert, *et al.*, The HADDOCK2.2 Web Server: User-Friendly Integrative Modeling of Biomolecular Complexes. *J Mol Biol* **428**, 720–725 (2016).

5. T. E. Exner, *et al.*, Binding of HasA by its transmembrane receptor HasR follows a conformational funnel mechanism. *Eur Biophys J* **49**, 39–57 (2020).

6. W. Humphrey, A. Dalke, K. Schulten, VMD: Visual molecular dynamics. *Journal of Molecular Graphics* **14**, 33–38 (1996).

7. J. Myers, G. Grothaus, S. Narayanan, A. Onufriev, A simple clustering algorithm can be accurate enough for use in calculations of pKs in macromolecules. *Proteins* **63**, 928–938 (2006).

8. J. C. Gordon, *et al.*, H++: a server for estimating pKas and adding missing hydrogens to macromolecules. *Nucleic Acids Research* **33**, W368–W371 (2005).

9. R. Anandakrishnan, B. Aguilar, A. V. Onufriev, H++ 3.0: automating pK prediction and the preparation of biomolecular structures for atomistic molecular modeling and simulations. *Nucleic Acids Research* **40**, W537–W541 (2012).

10. J. Beaufays, L. Lins, A. Thomas, R. Brasseur, In silico predictions of 3D structures of linear and cyclic peptides with natural and non-proteinogenic residues. *J Pept Sci* **18**, 17–24 (2012).

11. Z. Wang, J. Eickholt, J. Cheng, APOLLO: a quality assessment service for single and multiple protein models. *Bioinformatics* **27**, 1715–1716 (2011).

12. H. Kaur, A. Garg, G. P. S. Raghava, PEPstr: a de novo method for tertiary structure prediction of small bioactive peptides. *Protein Pept Lett* **14**, 626–631 (2007).

13. J. Maupetit, P. Derreumaux, P. Tufféry, A fast method for large-scale de novo peptide and miniprotein structure prediction. *J Comput Chem* **31**, 726–738 (2010).

14. J. Maupetit, P. Tuffery, P. Derreumaux, A coarse-grained protein force field for folding and structure prediction. *Proteins* **69**, 394–408 (2007).

15. A. C. Camproux, R. Gautier, P. Tufféry, A hidden markov model derived structural alphabet for proteins. *J Mol Biol* **339**, 591–605 (2004).

16. A. Sali, T. L. Blundell, Comparative protein modelling by satisfaction of spatial restraints. *J Mol Biol* **234**, 779–815 (1993).

17. R. Sánchez, A. Sali, Comparative protein structure modeling. Introduction and practical examples with modeller. *Methods Mol Biol* **143**, 97–129 (2000).

18. A. Fiser, A. Sali, ModLoop: automated modeling of loops in protein structures. *Bioinformatics* **19**, 2500–2501 (2003).

19. J. Lefèvre, P. Delepelaire, M. Delepierre, N. Izadi-Pruneyre, Modulation by substrates of the interaction between the HasR outer membrane receptor and its specific TonB-like protein, HasB. *J Mol Biol* **378**, 840–851 (2008).
