# Supplementary figures and images for: Dynamic interplay between a TonB-dependent heme transporter and a TonB protein in a membrane environment
Source: mBio. 2024 Oct 30;15(12):e01781-24. doi: 10.1128/mbio.01781-24 (PMC11633176; doi:10.1128/mbio.01781-24)

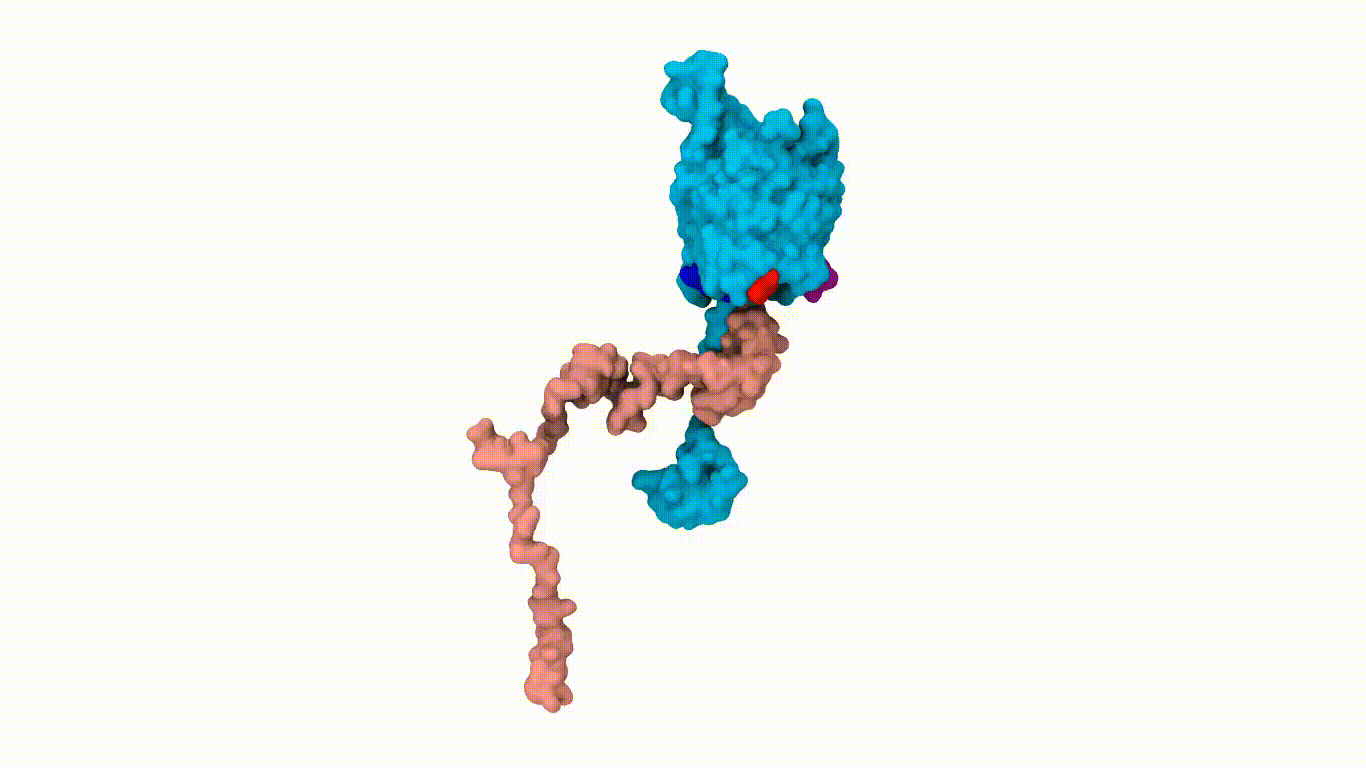

Supplement: Movie S1 — The video is taken from the first 120 ns of one of the HasR-HasB system simulations. [file mbio.01781-24-s0002.gif]
